# The Association between Dietary and Circulating Copper Levels and Osteoporosis: a Scoping Review

**DOI:** 10.1007/s12011-025-04899-1

**Published:** 2025-11-18

**Authors:** Ye Ju, Wei Xiong, Yuqi Pang, Zilan Chen, Jiaojiao Hou, Sirui Zheng, Zihao Li, Ting Liu, Hongxia Xia, Maoyao Xia, Yangdan Zhong, Jiayuan Li, Xia Jiang

**Affiliations:** 1 Department of Nutrition and Food Hygiene, West China School of Public Health and West China Fourth Hospital, Sichuan University, Chengdu, Sichuan China; 2 Department of Epidemiology and Biostatistics, West China School of Public Health and West China Fourth Hospital, Sichuan University, Chengdu, Sichuan China; 3 Department of Clinical Neuroscience, Karolinska Institute, Stockholm, Sweden; 4 West China School of Public Health and West China Fourth Hospital, Sichuan University, Chengdu, Sichuan, China

**Keywords:** Copper, Bone health, Osteoporosis, Fracture

## Abstract

Copper plays a crucial role in osteoporosis by regulating osteoblast function, inhibiting osteoclast activity, and promoting bone matrix maturation. However, the epidemiological relationship between copper and osteoporosis remains inconclusive. This scoping review systematically examined the existing evidence on the association between dietary and circulating copper levels and bone related diseases with a special focus on osteoporosis. Studies were identified through comprehensive search of electronic databases from inception to January 2025. Eligible studies included randomized clinical trials, cohort studies, case-control studies, and cross-sectional studies involving human subjects and published in English. The review included 18 studies. For dietary copper, studies reported that higher intake was associated with improved bone mineral density (BMD). For circulating copper, studies reported that lower levels were associated with higher subsequent risk of osteoporosis and fractures. For environmental copper, exposed via drinking water or hair copper levels were not associated with osteoporosis. Current evidence on the relationship between copper and bone health remains largely consistent, suggesting a potential inverse association between copper levels and osteoporosis risk.

**Supplementary Information:**

The online version contains supplementary material available at 10.1007/s12011-025-04899-1.

## Introduction

 Osteoporosis is a progressive skeletal disorder characterized by a decline in bone mineral density (BMD) and changes in bone turnover markers [1], which ultimately lead to weakened bone structure and increased risk of fracture [2, 3]. With the global aging population, the burden of osteoporosis and related complications are set to escalate [4], making it essential to explore cost-effective strategies for prevention and management. While multiple factors influence the development of osteoporosis [5], nutrition stands out as one of the most accessible and economical approaches to mitigate the risk of osteoporosis and subsequent fractures.

**Fig. 1:**
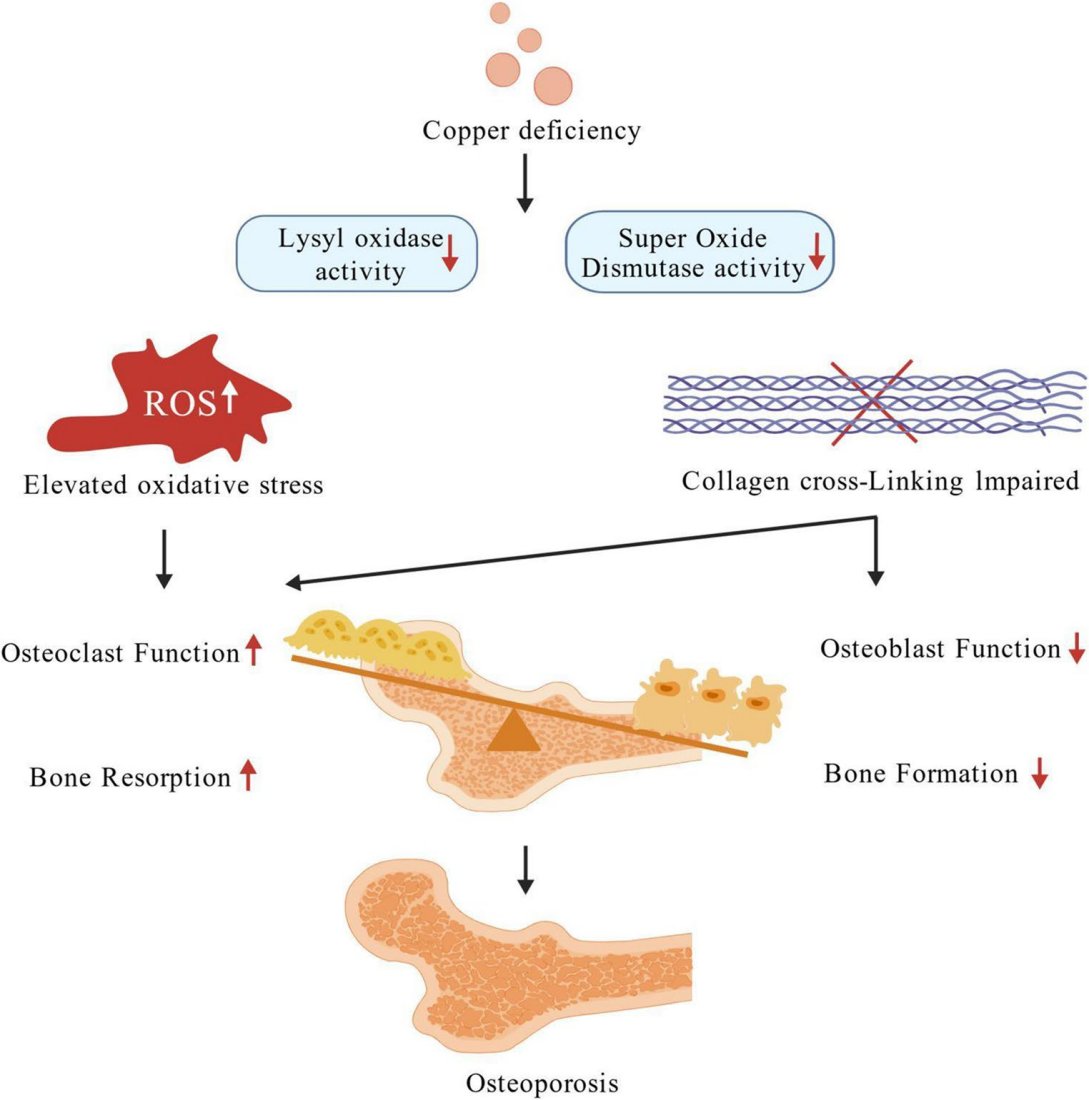
Possible biological mechanisms of copper deficiency in osteoporosis. (Created with BioGDP.com [12])

Copper is a vital trace element distributed across various tissues in human body and plays essential roles in maintaining skeletal integrity [6]. It influences bone metabolism through multiple pathways (Fig. 1). For example, copper regulates osteoblast and osteoclast activity, supports collagen synthesis and mineralization, and acts as a cofactor for lysyl oxidase [7]. It is also essential for antioxidant enzymes such as superoxide dismutase, of which deficiency leads to oxidative stress and impaired bone cell function, thereby causing increased activation of osteoclasts and higher levels of bone resorption, which ultimately contributes to the development of osteoporosis [8, 9]. Copper’s involvement in joint pathologies like osteoarthritis and rheumatoid arthritis further underscores its skeletal relevance [10, 11].

Experimental studies further support these mechanisms. For example, an experimental study based on SD rats showed that copper deficiency was associated with a decreased percentage of trabecular bone ranging from 35.79% to 25.06%, resulting in an increased risk for osteoporosis [13]. Despite the link between copper and osteoporosis being demonstrated at cellular level and supported by animal studies, findings from epidemiological research involving human populations remain inconsistent. For example, a cross-sectional study using data from NHANES indicated higher copper intake to be associated with an increased BMD in US adults. Specifically, participants in the highest quartile of copper intake (≥ 1.91 mg/day) had a mean total spine BMD that was 0.02 g/cm² greater than those in the lowest quartile (< 0.91 mg/day) [14]. However, in a case-control study of postmenopausal women conducted in Turkey, serum copper level did not significantly differ between osteoporosis cases and controls [15]. Notably, a meta-analysis including 5 studies of postmenopausal women demonstrated significantly lower circulating copper levels in osteoporotic patients compared to healthy controls. Yet this meta-analysis mixed studies of different designs and measurement approaches into one pooled analysis, of which heterogeneity restricted generalizability of findings. Taken together, despite consistent support from laboratory and animal-based observations, the translation of copper’s role on bone health into human populations remains complex and unresolved [16].

Therefore, the aim of this scoping review is to comprehensively examine and synthesize existing evidence on the impact of dietary copper as well as circulating copper on osteoporosis, its underlying biological measures (BMD, and bone turnover markers), and its worst clinical complication (fractures). By elucidating the potential sources of heterogeneity and by conducting an integrated analysis, we aim to clarify the role of copper in the onset and progression of osteoporosis and inform dietary recommendations and public health strategies aimed at reducing the burden of osteoporosis in an aging population.

## Methods

### Study Design

We performed a scoping review to identify the relationship of dietary copper, circulating and environmental copper with osteoporosis, providing insights into the potential role of copper in maintaining skeletal integrity and promoting bone health. We followed Arksey and O’Malley methodological framework [17] when developing this scoping review. This study was also conducted in accordance with PRISMA-ScR (the Preferred Reporting Items for Systematic Reviews and Meta-Analyses PRISMA guidelines with an extension for Scoping Reviews) [18].

### Search Strategy

To identify eligible articles for the scoping review, a comprehensive search strategy was performed including three databases (PubMed, Web of Science and Embase), from the inception of database to January 2025 following eligibility criteria. Our search strategy employed a comprehensive approach using both MeSH terms and free-text words across all databases. Briefly, it was built around key concepts including (“Copper”) AND (“Bone turnover*” OR “Bone density” OR “Bone fracture” OR “Osteoporosis”). The detailed search strategy is provided in Supplementary materials. This strategy was adapted and applied to the other two electronic databases in a similar manner.

### Eligibility Criteria

The inclusion criteria were:

1. Studies including participants recruited from diverse epidemiological sources, including samples from both hospitals and communities, without restriction on sex, age subgroup, or other demographic characteristics;
2. Participants aged ≥ 18 years;
3. Studies discussed osteoporosis including the disease itself, biological markers such as BMD or bone turnover markers, and clinical complication fracture;
4. Studies approved by an ethics committee.

The exclusion criteria were:

1. Not an epidemiological study;
2. Studies written not in English;
3. Studies not examined the effect of copper on either the onset or the progression of osteoporosis;
4. Studies that investigated multiple trace elements including copper, but did not assess the individual effect of copper;
5. Participants with severe systemic diseases (e.g., active malignant cancers) or known disorders of copper metabolism (e.g., Wilson’s disease);
6. Article types that were abstracts only, systematic reviews, letters to the editor, meta-analysis and opinion pieces.

### Study Selection and Data Collection Process

All retrieved records were initially exported into EndNote library (version X9, Clarivate, Philadelphia, USA). Subsequently, duplicate records were removed. The initial screening process involved evaluating titles and abstracts of the articles by two independent researchers (Ju and Chen), assessed against the pre-established eligibility criteria. In cases where discrepancies arose between the two researchers, a third researcher (Pang) was consulted to facilitate consensus. Following the initial screening, full texts of eligible articles were thoroughly analyzed by the same researchers, applying the eligibility criteria to ensure consistency and accuracy. Ultimately, full texts of the selected articles were systematically analyzed and evaluated by authors to serve the objectives of this scoping review.

### Data Extraction and Synthesis

Following the identification and finalization of eligible articles by the research team, a systematic data extraction process was conducted. Data were entered into a standardized extraction form designed in Microsoft Excel to ensure consistency and accuracy. The variables extracted encompassed the year of publication, country, study design, subject characteristics including sample size, age, description of intervention, methods of measurement on intervention, bone-related outcomes, methods of measurement on bone-related outcomes, and primary study findings. Descriptive statistics were generated to summarize the extracted data. Findings were presented through a narrative synthesis approach, which provided a comprehensive and cohesive overview of results, highlighting key trends and insights derived from the reviewed literature.

## Results

### Study Selection

**Fig. 2:**
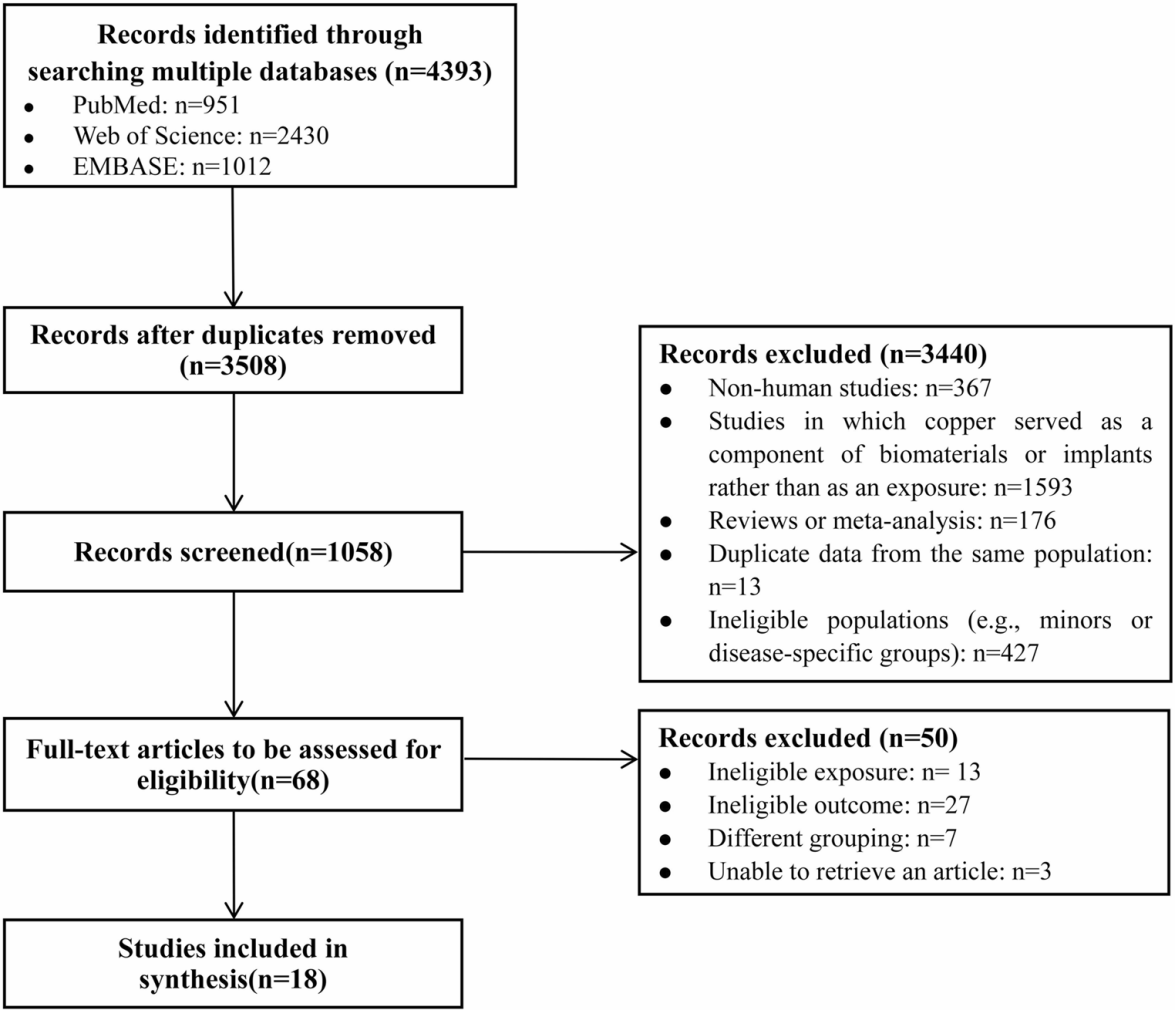
The flowchart of identification, screening and inclusion of studies

Flowchart shows the study selection process. Initially, 4,393 potentially relevant studies were identified through search in three databases. After removing duplicates, 3,508 papers were left. Following the screening of titles and abstracts, 3,440 studies were further excluded. Of the remaining 68 studies that underwent full-text screening based on eligibility criteria, only 18 were ultimately retained. A detailed selection process is depicted in Fig. 2.

### Study Characteristics

Table 1 details the study characteristics of the 18 studies, representing results from 14 cross-sectional studies (77.8%), 2 cohort studies (11%), 1 case-control study (5.6%) and 1 clinical trial (5.6%). Most of the studies were conducted in western countries (50%) followed by China (22.2%), Turkey (16.7%), Iran (11.1%). Altogether, 38,899 participants were involved. Excluding studies that did not provide sex ratio, the overall distribution of female-to-male ratio was 1.7: 1. A majority of participants were of middle-aged or elderly.

**Table 1:** Characteristics of included studies

NO.	First Author	Year	Country	Date of Collection, Survey/Recruitment	Study Design	Number of Subjects	Age
1	Julie A. Pasco [^19^]	2024	Australia	Geelong Osteoporosis Study	Cross-sectional	522 F	50.47 ± 22.36
2	Mingji Chen [^20^]	2024	USA	NHANES 2005–2014 and 2017–2018	Cross-sectional	5286 (F: M 2425:2861)	69.88 ± 0.13
3	Yonggang Fan [^14^]	2022	USA	NHANES 2007–2014 and 2017–2018	Cross-sectional	8224 (F: M 4300:3924)	47.76 ± 3.64
4	R. Abraham [^21^]	2006	UK	Primary care medical practices	Cohort	43 F	46–55 ^a^
5	A Baker [^22^]	1999	Ireland	Advertisement recruitment at the Norwich Research Park	Clinical trial	11 M	30.9 ± 11.2
6	Marjan Mahdavi-Roshan [^23^]	2015	Iran	The rheumatology clinic in Tabriz, Islamic Republic of Iran	Cross-sectional	51 F	61.98 ± 3.71
7	Naficeh Sadeghi [^24^]	2014	Iran	Bone mineral densitometry division of Jami Clinic	Cross-sectional	135 F	/
8	Shunzhi Liu [^25^]	2009	China	Postmenopausal women in Xi’an urban area.	Cross-sectional	290 F	45–65 ^a^
9	Muhong Wei [^26^]	2021	USA	NHANES2011-2016	Cross-sectional	2545 (F: M 1220:1325)	39.40 ± 0.40
10	Deniz Cemgil Arikan [^15^]	2011	Turkey	Department of Gynecology and Obstetrics of the Medical Faculty of Kahramanmaras Sutcu Imam	Case–control	107 F	54.70 ± 8.82
11	M Mutlu [^27^]	2007	Turkey	Orthopaedics Department of the Erciyes University Medical Faculty	Cross-sectional	120 F	43–80 ^a^
12	Muhong Wei [^28^]	2022	China	Physical Examination Center of Union Hospital of Tongji Medical College and two community health centers	Cross-sectional	627 (F: M 396:231)	≥ 50
13	Emre Okyay [^29^]	2013	Turkey	Division of Reproductive Endocrinology and Infertility, Department of Obstetrics and Gyne cology at Dokuz Eylul University School of Medicine	Cross-sectional	728 F	56.52 ± 6.14
14	D Conlan [^30^]	1990	England	Department of Orthopaedics, Glenfeld General Hospital	Cross-sectional	46 (Sex not revealed)	/
15	Xinhua Qu [^31^]	2018	USA	NHANES 2011–2014	Cross-sectional	722 (F: M 381:341)	56.47 ± 11.54
16	Cecilie Dahl [^32^]	2015	Norway	The NOREPOS Hip fracture database	Prospective cohort	18,926 (F: M 13493:5433)	50–85 ^a^
17	Wanli Li [^33^]	2005	China	People in Hongmen country of Xinxiang city.	Cross-sectional	470 (F: M 292:178)	65.82 ± 3.81
18	Zhixing Wang [^34^]	1987	China	Shanghai Institute of Traumatology and Orthopedics	Cross-sectional	25 (F: M 11:14)	14–85 ^a^

F Female; M Male; ^a^ Age data are presented as reported in original studies. For studies lacking mean age values, the age range is provided

Among the 18 studies included in the current review, 10 (55.6%) examined the role of circulating copper levels, 5 (27.8%) focused on dietary copper intake, 2 (11.1%) explored hair copper, and 1 (5.5%) investigated copper in drinking water. For outcomes, 11 studies focused on BMD, 4 studies focused on fracture, 3 studies focused on osteoporosis, and 1 study focused on bone metabolism biochemical markers. Methodological quality assessment of included studies and main results are proposed in Table 2.

**Table 2:** Main results of included studies

NO.	First Author	Outcomes	Measurement method of bone outcomes	Type of copper	Measurement method of copper	Results
1	Julie A. Pasco [^19^]	BMD	DXA	Dietary copper	A detailed semi-quantitative food frequency questionnaire	Low dietary intake of copper is associated with lower BMD at multiple skeletal sites in women
2	Mingji Chen [^20^]	BMD	DXA	Dietary copper	24-h dietary recall interviews	In elderly hypertensive patients, higher dietary copper intake is associated with increased BMD
3	Yonggang Fan [^14^]	BMD	DXA	Dietary copper	24-h dietary recall interviews	Higher dietary copper intake is linked to better bone health
4	R. Abraham [^21^]	BMD	DXA	Dietary copper	Weighed intake method	Positive association between copper intake and post-menopausal bone loss at the lumbar spine
5	A Baker [^22^]	Bone markers	ELISA	Copper supplementation	Laboratory test	No significant differences in serum osteocalcin levels between periods of low, medium, and high copper intake
6	Marjan Mahdavi-Roshan [^23^]	BMD	DXA	Serum copper	Laboratory test	Serum copper levels were significantly lower than the normal range in postmenopausal women with low bone density
7	Naficeh Sadeghi [^24^]	BMD	DXA	Plasma copper	Laboratory test	Plasma levels of copper in the osteoporotic women is higher than control, though the differences were not significant
8	Shunzhi Liu [^24^]	BMD	DXA	Serum copper	Laboratory test	No significant correlation between serum copper levels and BMD in postmenopausal women
9	Muhong Wei [^26^]	BMD	DXA	Serum copper	Laboratory test	Serum copper levels were not significantly associated with BMD in the overall analysis
10	Deniz Cemgil Arikan [^15^]	BMD	DXA	Serum copper	Laboratory test	Plasma copper levels were higher in osteoporotic patients than controls, but differences were not statistically significant
11	M Mutlu [^27^]	BMD	DXA	Serum copper	Laboratory test	No significant differences in serum copper levels between osteoporotic, osteopenic, and normal post-menopausal women
12	Muhong Wei [^28^]	Osteoporosis	DXA	Plasma copper	Laboratory test	No significant association between plasma copper levels and osteoporosis risk in Chinese adults aged ≥ 50 years
13	Emre Okyay [^29^]	Osteoporosis	DXA	Serum copper	Laboratory test	Low serum copper levels were significantly associated with postmenopausal osteoporosis, particularly in the early postmenopausal period
14	D Conlan [^30^]	Fracture	Hospital records	Serum copper	Laboratory test	Serum copper levels were significantly lower in elderly patients with femoral neck fractures compared to age and sex matched controls
15	Xinhua Qu [^31^]	Fracture	Self report	Serum copper	Laboratory test	Higher serum copper levels are significantly associated with an increased risk of total fracture, particularly in men
16	Cecilie Dahl [^32^]	Hip fracture	Hospital records	Water copper	Laboratory test	No association of copper levels in water with risk of hip fracture
17	Wanli Li [^33^]	BMD	DXA	Hair copper	Laboratory test	No significant correlation between hair copper levels and BMD in elderly individuals
18	Zhixing Wang [^34^]	Fracture	Hospital records	Hair copper	Laboratory test	Hair copper levels did not significantly change before and after fractures

BMD bone mineral density; DXA dual-emission X-ray absorptiometry; ELISA enzyme linked immunosorbent assay

### Dietary Copper Intake and Bone Health

A cross-sectional study conducted as part of the Geelong Osteoporosis Study by Pasco et al. [19] found low dietary copper intake to be associated with a decreased whole-body BMD in 522 women, after adjusting for age and weight (β = $\:-$0.021, SE = 0.006). This piece of finding was further supported by two other cross-sectional studies with enlarged sample size, both utilizing data from NHANES. Specifically, Chen et al. [20] analyzed data from 5,286 hypertensive participants and found each unit increase in dietary copper intake to be associated with an increased total femur BMD after adjusting for sociodemographic variables, behavioral characteristics and health factors (β = 0.101, 95%CI = 0.036–0.166). Similarly, Fan et al. [14] analyzed data from 8,224 participants and drew the same conclusion (β = 0.030, 95%CI = 0.020–0.040). In addition to the basic demographic characteristics, the study conducted by Fan et al. further adjusted for smoking status, hypertension, diabetes and the use of prednisone or cortisone. Similarly, a prospective cohort study conducted by Abraham et al. [21] in the UK followed 43 postmenopausal women for more than 10 (ranges 11–14) years, simple correlation analysis with no adjustment revealed a significant protective correlation of dietary copper intake on bone loss rate (*r* = $\:-$0.340, *P* < 0.05).

However, the protective correlation of dietary copper intake derived from observational studies was not supported by clinical trials. For example, Baker et al. [22] conducted a small-scale trial, following a total of 11 participants for merely 8 weeks, focusing on an intermediate outcome osteocalcin (rather than the disease osteoporosis or BMD). There was no significant difference in serum osteocalcin levels across different groups of low, medium or high copper intake (*P* > 0.05).

### Circulating Copper Levels and Bone Health

The initial study conducted by Mahdavi-Roshan et al. [23] examined serum copper levels in postmenopausal women with reduced BMD (*N* = 51). Findings showed that based on a simple group comparison, serum copper levels were significantly lower in the osteoporosis group than the osteopenia group (*P* < 0.001). However, this piece of evidence was not supported by subsequent studies of enlarged sample size. For example, Sadeghi et al. [24] analyzed data from 135 participants and found no statistically significant correlation between plasma copper concentrations and femoral BMD (*r* = $\:-$0.113, *P* = 0.072). Similarly, Liu et al. [25] involved 290 woman and demonstrated no significant correlation between serum copper levels and lumbar BMD (*r* = $\:-$0.036, *P* > 0.05). These null findings were further supported by a study conducted by Wei et al. [26] that included 2425 participants. After adjusting for demographic and clinical confounders, no association between serum copper levels and total BMD was found (β = 0.034, 95%CI = $\:-$0.075 ~ 0.008). Notably, all these four studies were of cross-sectional design. In line with their findings, a well-designed case-control study conducted by Arikan et al. (*N* = 107) [15] also reported a null association between plasma copper levels and femur BMD (*r* = $\:-$0.010, *P* > 0.05).

While BMD serves as a continuous indicator of osteoporosis, their clinical significance ultimately lies in the prediction and diagnosis of osteoporosis. Accordingly, several cross-sectional studies examined the relationship between circulating copper levels and osteoporosis. For instance, Mutlu et al. (*N* = 120) [27] observed no statistically significant differences with respect to serum copper levels between osteoporotic cases and normal controls (*P* > 0.05). Similarly, Wei et al. [28] involved 627 Chinese adults above 50 years old and drew the same conclusion (OR = 0.920, 95%CI = 0.570–1.470) after adjusting for demographic and clinical confounders. In contrast, Okyay et al. [29] involved 728 women and revealed lower serum copper levels to be associated with a significantly higher osteoporosis prevalence, after adjusting for duration of menopause and BMI (OR = 0.900, 95%CI = 0.960–0.980).

Fracture prevention remains the ultimate goal of osteoporosis management. Conlan et al. [30] reported in a study of small sample size (*N* = 46) that serum copper level was lower in patients with fractures compared to healthy controls (*P* < 0.01). Subsequently, Qu et al. [31] expanded this study to a sample size of 722. With adjustment of demographic variables, behavioral characteristics and lipid profile, a reduced serum copper levels were found to be associated with an elevated prevalence of fracture specifically in men (OR = 1.308, 95%CI = 1.025–1.670).

### Environmental Copper Exposure and Bone Health

Beyond endogenous exposure, such as dietary intake and circulating levels, increasing attention has been directed towards exogenous exposure through environmental pathways. For water copper, Dahl et al. [32] conducted a prospective cohort study as part of the Norwegian Epidemiologic Osteoporosis Studies and found no association of copper levels in water with hip fracture after adjustment (IRR = 1.050, 95%CI = 0.960–1.140). Similarly, for hair copper, Li et al. [33] revealed no significant correlation with BMD (*r* = 0.048, *P* > 0.05). Wang et al. [34] also found no significant difference before and after fractures by group comparison (*P* > 0.05).

## Discussion

The purpose of this scoping review was to identify and examine the existing literature regarding the relationship between dietary, circulating copper and the onset and progression of osteoporosis. Based on 18 eligible studies, insufficient dietary copper intake and low circulating copper levels are implicated in the onset and progression of osteoporosis.

The association between copper and bone health observed in this review aligns with a broader pattern regarding the involvement of trace element in skeletal metabolism. Not only copper, but also other essential metals such as zinc and iron have been implicated in osteoporosis pathogenesis through overlapping biological mechanisms, including antioxidant activity, regulation of bone cell differentiation, and collagen formation. For instance, a cohort study based on 4,924 participants from the Dongfeng-Tongji cohort found that plasma zinc levels were positively associated with an increased prevalence of osteoporosis in females [35]. A cross-sectional study involving 143 participants found a significant higher serum iron concentration in fracture group compared to non-fracture group (*P* < 0.05) [36]. These findings suggest that similar to copper, circulating trace elements play a vital role in maintaining bone health.

Studies included in this scoping review showed significant heterogeneity in terms of design, exposure assessment, and participant characteristics. Notably, cross-sectional studies are limited in their ability to infer temporal or causal relationships whereas clinical trials provide more robust evidence. Unfortunately, the only clinical trial [37] we identified in our scoping review did not confirm a significant association, likely due to its small sample size that yielded to limited statistical power. Moreover, the absence of randomization of group allocation as well as the short intervention period of 8 weeks might further preclude meaningful changes at osteocalcin levels. Unlike previous studies that assessed BMD, this trial used a bone turnover marker as primary outcome. It is likely that changes in other pathways related to bone resorption or mineralization may not be reflected at osteocalcin levels alone [37].

A key source of heterogeneity among studies lies in the methods used to assess copper exposure. Specifically, dietary copper intake was assessed through 24-hour recalls, self-developed FFQs, and direct food weighing. These methods vary in accuracy and are subject to different forms of bias. Similarly, circulating copper was assessed using different analytical techniques for which variation existed in fasting duration, sample type, and blood processing protocols. These inconsistencies may introduce intra-method variability and measurement error, thereby complicate interpretation and potentially obscuring true associations. This highlights the urgent need for standardized, validated methodologies in future. Our study investigating the link between environmental copper exposure and bone health has in general produced negative findings. These null findings could be due to the relatively low levels of environmental copper exposure compared to endogenous sources. It is possible that such levels of copper found in environmental sources may not be sufficient to significantly alter bone metabolism, especially when considering the complex nature of bone turnover and the multiple factors influencing BMD and fracture.

Despite considerable heterogeneity across studies, the association between copper status and bone health remained generally consistent across various subpopulations. Specifically, this relationship was observed in postmenopausal women, who face a heightened risk of osteoporosis due to estrogen deficiency and accelerated bone turnover, as well as among individuals with hypertension. The reproducibility of association across groups with distinct demographic and clinical characteristics suggests the relationship is unlikely to depend on specific features but rather reflects a generalizable population-wide association. Although the precise biological mechanisms remain to be fully elucidated, existing evidence indicates that copper contributes to bone metabolism through processes such as collagen cross-linking and antioxidant defense [38, 39]. Taken together, these findings highlight the potential importance of maintaining adequate copper status for skeletal health across diverse adult populations, which may inform strategies aimed at osteoporosis prevention.

When the focus was shifted from BMD or osteoporosis to its clinical complication fractures, serum copper remained as a potential risk factor with an even more pronounced effect. This pattern suggests that the impact of copper on bone health may become more apparent as the situation deteriorates, underscoring its complexity in bone metabolism. An intriguing question is why the effects of copper become more evident during the stage of clinical complications. Copper is an essential cofactor for lysyl oxidase, an enzyme required for the cross-linking of collagen fibers that are fundamental to the structural integrity of bone matrix. Impaired collagen maturation due to copper deficiency can compromise bone quality and mechanical strength even in the presence of normal BMD [38], thus increasing susceptibility to fractures. In addition, copper contributes to antioxidant defense as a key component of copper-zinc superoxide dismutase [39]. These functions suggest that copper may play a more critical role in maintaining bone integrity under pathological rather than physiological conditions.

From a clinical perspective, our findings suggest that copper status - assessed via dietary intake or circulating levels - may be considered as one of several factors in comprehensive osteoporosis risk stratification, particularly among high-risk populations such as postmenopausal women. Therefore, ensuring sufficient dietary copper intake represents a practical and potentially modifiable approach that may contribute to bone health, although this interpretation should be viewed with caution given the limited interventional and longitudinal evidence currently available.

From a public health perspective, encouraging sufficient copper intake as part of balanced nutrition for bone health may be valuable. Although such recommendations should be made cautiously given the largely observational and heterogeneous nature of the current evidence, these findings nonetheless provide a useful basis for developing targeted educational initiatives and integrating copper nutrition into broader public health strategies for osteoporosis and fracture prevention.

### Strengths and Limitations

Our study adhered to rigorous methodological frameworks to ensure a transparent and clear review process. A key strength of this review is its exclusive focus on primary research, which allowed us to directly map the foundational literature and provide an unbiased overview of original studies on copper and bone health. Other limitations must also be noted. Considerable heterogeneity among the included studies, especially in design, population characteristics, and measurement methods, restricted the potential for direct comparison and synthesis of results. In addition, measurement inaccuracies in copper assessment, along with varying control for confounding factors across studies, may also compromise the accuracy and reliability of the findings and help explain the inconsistencies observed.

### Future Research

Future research on copper’s role in osteoporosis should focus on standardizing the assessment of both dietary and circulating copper to reduce heterogeneity and improve comparability across studies. Cohorts with long term follow up and interventional studies are needed to evaluate the sustained impact of copper on osteoporosis and to quantitatively define the dose-response relationship, thereby determining the optimal range of copper exposure that is both safe and beneficial for long-term bone health. These studies should include diverse populations across different ages, sexes, health statuses, regions, and dietary habits to determine the universal applicability of copper’s potential benefits on osteoporosis.

## Conclusion

Evidence surrounding dietary copper intake, circulating and environmental copper levels suggests that a higher level of copper may have a beneficial impact on bone health including BMD, osteoporosis and fractures prevention. However, the current evidence is insufficient to delineate a clear dose-response relationship. It remains unclear whether this relationship is linear or U-shaped, and consequently what constitutes an optimal or safe range of copper exposure. These findings underscore the need to develop standardized biomarkers for assessing copper status as well as the importance of conducting long-term interventional studies to establish the efficacy of copper supplementation. Specifically, future research should prioritize elucidating the dose-response relationship to define a safe and effective range of copper exposure for bone health. Advancing these research directions could support the development of evidence-based strategies to incorporate copper monitoring and supplementation into public health initiatives for osteoporosis prevention.

## Supplementary Information

Below is the link to the electronic supplementary material.

ESM 1 DOCX(14.5 KB)

## Data Availability

No datasets were generated or analysed during the current study.
