# Supplementary material for: The Association between Dietary and Circulating Copper Levels and Osteoporosis: a Scoping Review
Source: Biol Trace Elem Res. 2025 Nov 18;204(5):3366–75. doi: 10.1007/s12011-025-04899-1 (PMC13149565; doi:10.1007/s12011-025-04899-1)
Supplement: Supplementary file 1 — DOCX(14.5 KB) [file 12011_2025_4899_MOESM1_ESM.docx]

**Supplementary Table. Search strategy of The Association Between Dietary and Circulating Copper Levels and Osteoporosis: A Scoping Review**

PubMed

| Search | Query |
| --- | --- |
| #1 | ((Osteoporosis[MeSH Terms]) OR (Osteoporoses[Title/Abstract] OR Osteoporosis, Age-Related[Title/Abstract] OR Osteoporosis, Age Related[Title/Abstract] OR Age-Related Osteoporosis[Title/Abstract] OR Age-Related Osteoporoses[Title/Abstract] OR Age Related Osteoporosis[Title/Abstract] OR Osteoporoses, Age-Related[Title/Abstract] OR Bone Loss, Age-Related[Title/Abstract] OR Age-Related Bone Loss[Title/Abstract] OR Age-Related Bone Losses[Title/Abstract] OR Bone Loss, Age Related[Title/Abstract] OR Bone Losses, Age-Related[Title/Abstract] OR Osteoporosis, Senile[Title/Abstract] OR Osteoporoses, Senile[Title/Abstract] OR Senile Osteoporoses[Title/Abstract] OR Senile Osteoporosis[Title/Abstract] OR Osteoporosis, Involutional[Title/Abstract] OR Osteoporosis, Post-Traumatic[Title/Abstract] OR Osteoporosis, Post Traumatic[Title/Abstract] OR Post-Traumatic Osteoporoses[Title/Abstract] OR Post-Traumatic Osteoporosis[Title/Abstract]) OR (Osteoporosis, Postmenopausal[MeSH Terms]) OR (Osteoporosis, Post-Menopausal[Title/Abstract] OR Osteoporoses, Post-Menopausal[Title/Abstract] OR Osteoporosis, Post Menopausal[Title/Abstract] OR Post-Menopausal Osteoporoses[Title/Abstract] OR Post-Menopausal Osteoporosis[Title/Abstract] OR Postmenopausal Osteoporosis[Title/Abstract] OR Osteoporoses, Postmenopausal[Title/Abstract] OR Postmenopausal Osteoporoses[Title/Abstract] OR Perimenopausal Bone Loss[Title/Abstract] OR Bone Loss, Postmenopausal[Title/Abstract] OR Bone Losses, Postmenopausal[Title/Abstract] OR Postmenopausal Bone Losses[Title/Abstract] OR Postmenopausal Bone Loss[Title/Abstract] OR Bone Loss, Perimenopausal[Title/Abstract] OR Bone Losses, Perimenopausal[Title/Abstract] OR Perimenopausal Bone Losses[Title/Abstract])) AND (copper) |
| #2 | ((sarcopenia[MeSH Terms]) OR (Sarcopenia*[Title/Abstract])) AND (copper) |
| #3 | ((Bone Fracture[Title/Abstract] OR Fracture, Bone[Title/Abstract] OR Bone Fractures[Title/Abstract] OR Broken Bones[Title/Abstract] OR Bone, Broken[Title/Abstract] OR Bones, Broken[Title/Abstract] OR Broken Bone[Title/Abstract] OR Spiral Fractures[Title/Abstract] OR Fracture, Spiral[Title/Abstract] OR Fractures, Spiral[Title/Abstract] OR Spiral Fracture[Title/Abstract] OR Torsion Fractures[Title/Abstract] OR Fractures, Torsion[Title/Abstract] OR Fracture, Torsion[Title/Abstract] OR Torsion Fracture[Title/Abstract]) OR ("Fractures, Bone"[Mesh])) AND (copper) |
| #4 | ((bone density[MeSH Terms]) OR (Bone Densities[Title/Abstract] OR Density, Bone[Title/Abstract] OR Bone Mineral Density[Title/Abstract] OR Bone Mineral Densities[Title/Abstract] OR Density, Bone Mineral[Title/Abstract] OR Bone Mineral Content[Title/Abstract] OR Bone Mineral Contents[Title/Abstract] OR BMD[Title/Abstract])) AND (copper) |
| #5 | ("alanine phosphatase"[Title/Abstract] OR "bone alkaline phosphatase"[Title/Abstract] OR "osteocalcin"[Title/Abstract] OR "parathyroid hormone"[Title/Abstract] OR "bone turnover*"[Title/Abstract]) AND (copper) |

EMBASE

| Search | Query |
| --- | --- |
| #1 | (Osteoporosis or age related osteoporosis or age-related bone loss or age-related bone losses or age-related osteoporoses or bone loss, age related or bone losses, age-related or osteoporoses or osteoporoses, age-related or osteoporoses, senile or osteoporosis or osteoporosis, age related or osteoporosis, involutional or osteoporosis, post traumatic or osteoporosis, senile or post-traumatic osteoporoses or post-traumatic osteoporosis or senile osteoporoses or senile osteoporosis).ti. or (Osteoporosis or age related osteoporosis or age-related bone loss or age-related bone losses or age-related osteoporoses or bone loss, age related or bone losses, age-related or osteoporoses or osteoporoses, age-related or osteoporoses, senile or osteoporosis or osteoporosis, age related or osteoporosis, involutional or osteoporosis, post traumatic or osteoporosis, senile or post-traumatic osteoporoses or post-traumatic osteoporosis or senile osteoporoses or senile osteoporosis).ab. |
| #2 | copper.ti. or copper.ab. |
| #3 | (Osteoporosis, Postmenopausal or bone loss, perimenopausal or bone loss, postmenopausal or bone losses, perimenopausal or bone losses, postmenopausal or osteoporoses, post-menopausal or osteoporosis, post menopausal or perimenopausal bone loss or perimenopausal bone losses or post-menopausal osteoporoses or post-menopausal osteoporosis or postmenopausal bone loss or postmenopausal bone losses).ti. or (Osteoporosis, Postmenopausal or bone loss, perimenopausal or bone loss, postmenopausal or bone losses, perimenopausal or bone losses, postmenopausal or osteoporoses, post-menopausal or osteoporosis, post menopausal or perimenopausal bone loss or perimenopausal bone losses or post-menopausal osteoporoses or post-menopausal osteoporosis or postmenopausal bone loss or postmenopausal bone losses).ab. |
| #4 | 1 or 3 |
| #5 | 2 and 4 |
| #6 | (fracture or bone cement fracture or bone fracture or bone fractures or broken bone or broken bones or closed fracture or closed fractures or fractured bone or fractures or fractures, bone or fractures, closed or skeleton fracture or unstable fracture Osteoporosis fracture or fracture, osteoporotic or fractures, osteoporotic or osteoporotic fracture or osteoporotic fractures).ti. or (fracture or bone cement fracture or bone fracture or bone fractures or broken bone or broken bones or closed fracture or closed fractures or fractured bone or fractures or fractures, bone or fractures, closed or skeleton fracture or unstable fracture Osteoporosis fracture or fracture, osteoporotic or fractures, osteoporotic or osteoporotic fracture or osteoporotic fractures).ab. |
| #7 | (sarcopenia or sarcopenia or sarcopenias).ti. or (sarcopenia or sarcopenia or sarcopenias).ab. |
| #8 | (bone density or bone mineral density or density, bone or osseous density).ti. or (bone density or bone mineral density or density, bone or osseous density).ab. |
| #9 | 2 and 8 |
| #10 | (bone turn-over or osseous turnover or alanine phosphatase or bone alkaline phosphatase or osteocalcin or parathyroid hormone or bone turnover).ti. or (bone turn-over or osseous turnover or alanine phosphatase or bone alkaline phosphatase or osteocalcin or parathyroid hormone or bone turnover).ab. |
| #11 | 2 and 10 |
| #12 | 2 and 6 |

Web of Science

| Search | Query |
| --- | --- |
| #1 | bone density OR Bone Densities OR Bone Mineral Density OR Bone Mineral Densities OR Bone Mineral Content OR Bone Mineral Contents OR BMD (Title) OR bone density OR Bone Densities OR Bone Mineral Density OR Bone Mineral Densities OR Bone Mineral Content OR Bone Mineral Contents OR BMD (Abstract) and Preprint Citation Index (Exclude – Database) |
| #2 | Osteoporosis OR Osteoporoses OR Age-Related Osteoporosis OR Age-Related Osteoporoses OR Age Related Osteoporosis OR Age-Related Bone Loss OR Age-Related Bone Losses OR Senile Osteoporoses OR Senile Osteoporosis OR Post-Traumatic Osteoporoses OR Post-Traumatic Osteoporosis (Title) OR Osteoporosis OR Osteoporoses OR Age-Related Osteoporosis OR Age-Related Osteoporoses OR Age Related Osteoporosis OR Age-Related Bone Loss OR Age-Related Bone Losses OR Senile Osteoporoses OR Senile Osteoporosis OR Post-Traumatic Osteoporoses OR Post-Traumatic Osteoporosis (Abstract) and Preprint Citation Index (Exclude – Database) |
| #3 | Bone Fracture OR Fracture, Bone OR Bone Fractures OR Broken Bones OR Bone, Broken OR Bones, Broken OR Broken Bone OR Spiral Fractures OR Fracture, Spiral OR Fractures, Spiral OR Spiral Fracture OR Torsion Fractures OR Fractures, Torsion OR Fracture, Torsion OR Torsion Fracture OR Osteoporosis fracture OR Osteoporotic Fracture (Title) OR Bone Fracture OR Fracture, Bone OR Bone Fractures OR Broken Bones OR Bone, Broken OR Bones, Broken OR Broken Bone OR Spiral Fractures OR Fracture, Spiral OR Fractures, Spiral OR Spiral Fracture OR Torsion Fractures OR Fractures, Torsion OR Fracture, Torsion OR Torsion Fracture OR Osteoporosis fracture OR Osteoporotic Fracture (Abstract) and Preprint Citation Index (Exclude – Database) |
| #4 | Osteopenia (Title) OR Osteopenia (Abstract) and Preprint Citation Index (Exclude – Database) |
| #5 | alanine phosphatase OR bone alkaline phosphatase OR osteocalcin OR parathyroid hormone OR bone turnover OR bone turnover marker (Title) OR alanine phosphatase OR bone alkaline phosphatase OR osteocalcin OR parathyroid hormone OR bone turnover OR bone turnover marker (Abstract) and Preprint Citation Index (Exclude – Database) |
| #6 | copper (Title) OR copper (Abstract) and Preprint Citation Index (Exclude – Database) |
| #7 | #1 AND #6 and Preprint Citation Index (Exclude – Database) |
| #8 | #2 AND #6 and Preprint Citation Index (Exclude – Database) |
| #9 | #3 AND #6 and Preprint Citation Index (Exclude – Database) |
| #10 | #4 AND #6 and Preprint Citation Index (Exclude – Database) |
| #11 | #5 AND #6 and Preprint Citation Index (Exclude – Database) |
